# Genetic structure of the population of wild-growing vines of the Utrish Nature Reserve

**DOI:** 10.18699/VJGB-23-38

**Published:** 2023-07

**Authors:** E.T. Ilnitskaya, M.V. Makarkina, I.V. Gorbunov, I.V. Stepanov, T.D. Kozina, E.A. Kozhevnikov, V.K. Kotlyar

**Affiliations:** North Caucasian Federal Scientific Center of Horticulture, Viticulture, Wine-making, Krasnodar, Russia; North Caucasian Federal Scientific Center of Horticulture, Viticulture, Wine-making, Krasnodar, Russia; Anapa Zonal Experimental Station of Viticulture and Wine-making – Branch of North Caucasian Federal Scientific Center of Horticulture, Viticulture, Wine-making, Anapa, Russia; North Caucasian Federal Scientific Center of Horticulture, Viticulture, Wine-making, Krasnodar, Russia; North Caucasian Federal Scientific Center of Horticulture, Viticulture, Wine-making, Krasnodar, Russia; North Caucasian Federal Scientific Center of Horticulture, Viticulture, Wine-making, Krasnodar, Russia; North Caucasian Federal Scientific Center of Horticulture, Viticulture, Wine-making, Krasnodar, Russia

**Keywords:** wild-growing vines, Vitis sylvestris, DNA profiling, genetic polymorphism, SSR loci, дикорастущие формы винограда, Vitis sylvestris, ДНК-профилирование, генетический полиморфизм, SSR-локусы

## Abstract

Grapes are one of the most common agricultural crops in the world. Currently, the analysis of genotypes directly at the DNA level is considered to be the most accurate method for studying the plant gene pool. The study of wild vines and ancient varieties in various regions of viticulture is an important direction of research in this field. The purpose of this work was to study the population of wild grapes growing on the territory of the Utrish Nature Reserve on the Black Sea coast of Krasnodar Region. The territory of the reserve is of interest as it is a site of ancient settlements, and the environmental conditions are suitable for the growth of wild grapes. During the survey of the territory, 24 samples of wild grapes were found, which were described according to the main morphological characteristics and analyzed by the molecular genetic method. The found vines were genotyped using 15 DNA markers, including nine commonly used for DNA fingerprinting (VVS2, VVMD5, VVMD7, VVMD25, VVMD27, VVMD28, VVMD32, VrZAG62, VrZAG79) and VVIb23, which allows determining hermaphrodite and dioecious vines. Statistical processing of microsatellite loci polymorphism data was carried out using the GenAlEx 6.5 program. The genetic relationships of the studied vines were evaluated using the PAST 2.17c program. The samples were found to be morphologically and genetically polymorphic. The number of alleles identified in the sample varied from 5 to 18 and averaged 8 alleles per locus. Statistical processing of DNA analysis data made it possible to identify two genetically different populations among the wild discovered vines. An assessment of genetic similarity of the found vines with some local varieties of geographically close viticulture regions, rootstocks and representatives of Vitis sylvestris from other territories was made. One of the populations found in the Utrish Nature Reserve is close to a number of V. sylvestris genotypes, the DNA profiles of which are presented in the Vitis International Variety Catalogue.

## Abstract (ru)

Виноград – одна из самых распространенных сельскохозяйственных культур в мире. В настоящее время наиболее точным методом для изучения генофонда растений считается анализ генотипов на уровне ДНК. Дикорастущие формы и древние сорта различных регионов виноградарства – актуальные объекты исследований в данной области. Целью работы было изучение популяции дикорастущего винограда, произрастающей на территории заповедника «Утриш» на Черноморском побережье Краснодарского края. Территория заповедника представляет интерес для подобного рода исследований, так как является местом древних поселений, а экологические условия пригодны для произрастания диких форм винограда. В процессе обследования территории обнаружено 24 образца дикорастущего винограда, которые были описаны по основным морфологическим характеристикам и проанализированы молекулярно-генетическим методом. Найденные формы генотипированы с помощью 15 ДНК-маркеров, в том числе девяти общепринятых для ДНК-паспортизации сортов винограда (VVS2, VVMD5, VVMD7, VVMD25, VVMD27, VVMD28, VVMD32, VrZAG62, VrZAG79) и маркера VVIb23, позволяющего определять обоеполые и двудомные формы. Статистическая обработка данных полиморфизма микросателлитных локусов выполнена в программе GenAlEx 6.5. Генетические взаимосвязи исследуемых форм винограда оценивали в программе PAST 2.17c. В результате обнаружено, что образцы характеризуются морфологическим и генетическим полиморфизмом. Количество идентифицированных аллелей в выборке варьировало от 5 до 18 и составило в среднем 8 аллелей на локус. Проведение статистической обработки данных ДНК-анализа позволило выявить две генетически различные популяции среди обнаруженных дикорастущих форм. Оценено генетическое сходство найденных форм с некоторыми аборигенными сортами географически близких регионов виноградарства, подвойными сортами и представителями Vitis sylvestris с других территорий. Одна из обнаруженных в заповеднике «Утриш» популяций близка к ряду генотипов V. sylvestris, ДНК-профили которых представлены в международной базе данных Vitis International Variety Catalogue.

## Introduction

Grapes are one of the most widespread agricultural crops in the world. The most significant, both economically and sociohistorically, is the species Vitis vinifera L. This species of the genus Vitis L. (family Vitaceae), originating from Eurasia, supposedly appeared about 65 million years ago (This et al., 2006). Currently, two subspecies are distinguished within the species, V. vinifera L. subsp. sylvestris (Gmel.) Hegi, which includes wild populations, and V. vinifera subsp. sativa (DC.) Hegi (or subsp. vinifera), which includes cultivars.

Wild and cultivated vines differ in a number of features, including their reproductive biology: wild grapevines are dioecious and cross-pollinate, while the cultivated vines are mostly hermaphrodite and self-pollinating. The domestication of grapes, which occurred about 8 thousand years ago, is closely associated with the emergence of winemaking, although it is still not known for certain which process preceded the other. The Middle East and the Caucasus are considered to be the initial centers of domestication of V. vinifera. The earliest evidence of wine production from 7,400–7,000 BC is found in Iran (McGovern, 2004). Seeds of domesticated grapes, about 8,000 years old, have also been found in Georgia and Turkey. However, Neolithic seed remains found in Western Europe also suggest grape exploitation during this time, and wild form seed remains have been found at Bronze Age sites in France (This et al., 2006).

Wild grape populations are currently represented by wild vines of V. vinifera cultivars and a wild subspecies. The study of wild grapevines in the ancient regions of grape cultivation has been actively conducted in recent years at the molecular genetic level (Doulati-Baneh et al., 2015; Gorislavec et al., 2017; De Michele et al., 2019; Margaryan et al., 2019; Cunha et al., 2020; Zdunić et al., 2020; Kupe et al., 2021; Lukšić et al., 2022).

The study of the local gene pools of various viticulture regions (including native varieties and wild specimens) at the DNA level makes it possible to more fully assess the genetic diversity of varieties and vines, to identify closer and more distant genotypes. Wild exemplars of agricultural crops are also significant for breeding as unique sources of genetic variability (Ellstrand et al., 2010).

The territory of the Utrish Nature Reserve on the Black Sea coast of the Krasnodar Territory is of interest for this kind of research, since it is a site of ancient settlements, and the ecological conditions of the territory are suitable for the growth of wild vines (Chernodubov, Rudenok, 2015).

## Materials and methods

Expeditionary research to search for wild grapevines, study the ecological conditions of their habitats and morpho-biological features was carried out for three years (2019–2021) on the territory of the Utrish Nature Reserve (Krasnodar Territory). The reserve is located in the northwestern part of the Black Sea coast of the Western Caucasus, on the Abrau Peninsula. The climate is sub-Mediterranean, moderately warm.

For molecular genetic analysis, 24 samples of wild-growing grapes were selected. DNA samples were isolated from shoot apical parts of vine plants by a method based on the use of CTAB (cytyltrimethylammonium bromide) (Rogers, Bendich, 1985).

Genotyping was performed at 15 microsatellite loci, 9 of which are standard for DNA fingerprinting of grape varieties (VVS2, VVMD5, VVMD7, VVMD25, VVMD27, VVMD28, VVMD32, VrZAG62, VrZAG79) (This et al., 2004; This, 2007). DNA markers linked to grape pathogens resistance loci were also included in the study – downy mildew resistance: UDV305, UDV737 (Rpv3) and GF09-46 (Rpv10) and powdery mildew resistance: ScORGF15-02 (Ren3), CenGen6 (Ren9) (Di Gaspero et al., 2012; Schwander et al., 2012; van Heerden et al., 2014; Zendler et al., 2017). All found specimens were also analyzed with the VVIb23 marker, which makes it possible to detect hermaphrodite and dioecious samples (Merdinoglu et al., 2005; Riaz et al., 2013).

Polymerase chain reaction (PCR) was carried out using an Eppendorf MasterCycler nexus GX2 device (Germany) according to the following scheme: 5 minutes at +95 °С (initial denaturation); 35 cycles: 10 seconds at +95 °С (denaturation), primer annealing for 30 seconds at +55 °С for VVS2, VVMD5, VVMD7, VVMD27, UDV305, UDV737, CenGen6, VVIb23, at +58 °С for VrZAG62, VrZAG79, ScORGF15-02, at +60 °С for VVMD25, VVMD28, VVMD32, GF09-46, 30 seconds at +72 °C (elongation); the final cycle was 15 minutes at +72 °С. PCR mixture with a total volume of 20 μl contained: 50 ng of genomic DNA, 1.5 units of Taq polymerase, 1x buffer for Taq polymerase with ammonium sulfate and magnesium, 2 mM MgCl2, 0.2 mM of each dNTP (deoxynucleotide triphosphates) (SibEnzyme-M, Moscow) and 200 μM of each of the primers (Sintol, Moscow). The results were visualized by capillary electrophoresis using a Nanofor 05 genetic analyzer (Institute of Analytical Instrumentation, Russian Academy of Sciences, St. Petersburg, Russia) and a special built-in software package.

The following varieties were used as reference genotypes: Pinot noir (for markers VVS2, VVMD5, VVMD7, VVMD25, VVMD27, VVMD28, VVMD32, VrZAG62, VrZAG79), Saperavi Severniy (GF09-46), Villard blanc (UDV305, UDV737), Regent (ScORGF15-02, CenGen6), Kishmish Vatkana (VVIb23), since the allelic compositions in the DNA of these varieties for the analyzed loci are known.

Statistical processing of microsatellite loci polymorphism data was carried out using the GenAlEx 6.5 program (Peakall, Smouse, 2012). The genetic relationships of the studied grapevines were assessed using the PAST 2.17c program using the method of principal coordinates (PCoA) (Hammer et al., 2001).

In order to study the genetic similarity of the studied wildgrowing samples with the indigenous gene pool of grapes, the work included the sampling of DNA profiles of cultivars according to nine standard SSR loci from the international database Vitis International Variety Catalog (VIVC), which belong to the local forms of Dagestan (Republic of Dagestan, Russian Federation), Don (Rostov region, Russian Federation), Georgia, Crimea (Republic of Crimea, Russian Federation), as well as to rootstock grape cultivars (the greatest contribution to the genotype of which was made by different North American species) and V. sylvestris genotypes from different geographical zones. Bayesian analysis was carried out in the Structure 2.3.4 program using 65 genotypes (41 varieties from the VIVC database and 24 genotypes of the studied forms) with the following parameters: 500000 Burn-in period, 500000 Reps, K=7 (Pritchard et al., 2000).

The study was carried out using the instrument park of the Center for Collective Use of Technological Equipment in the direction of “Genomic and postgenomic technologies” of the North Caucasian Federal Scientific Center for Horticulture, Viticulture, Wine-making.

## Results and discussion

In the process of surveying the territory of the Utrish Nature Reserve, 24 samples of wild-growing grapes were found, the description of which according to the main morphological features is given in Table 1.

**Table 1.**
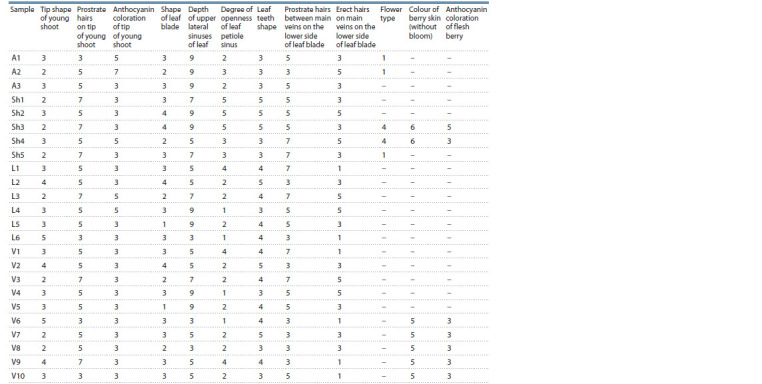
Morphological characteristics of wild-growing vines, Utrish Nature Reserve Notе. Tip shape of young shoot: 2 – slightly open; 3 – half open; 4 – wide open; 5 – fully open. Prostrate hairs on tip of young shoot: 3 – sparse; 5 – medium; 7 – dense. Anthocyanin coloration of tip of young shoot: 3 – weak; 5 – medium; 7 – strong. Shape of leaf blade: 1 – cordate; 2 – wedge-shaped; 3 – pentagonal; 4 – circular. Depth of upper lateral sinuses of leaf: 3 – shallow; 5 – medium; 7 – deep; 9 – very deep. Degree of openness of leaf petiole sinus: 1 – very wide open; 2 – wide open; 3 – open, 4 – slightly open; 5 – closed. Leaf teeth shape: 3 – both sides convex; 4 – one side concave, one side convex; 5 – mixture of both sides straight and both sides convex. Prostrate hairs between main veins on the lower side of leaf blade: 3 – sparse; 5 – medium; 7 – dense. Erect hairs on main veins on the lower side of leaf blade: 1 – absent or very sparse; 3 – sparse; 5 – medium. Flower type: 1 – male; 4 – female. Colour of berry skin (without bloom): 5 – dark red-violet; 6 – blue black. Anthocyanin coloration of flesh berry: 3 – weak; 5 – medium. Dash – no data available.

Polymorphism of morphological traits was noted both between populations from different sampling sites, and between plants within nominal populations, designated by us according to their places of growth on the territory of the reserve (plants were found in the locations of Atmacheva Shchel (A1–A3), Shirokaya Shchel (Sh1–Sh5), Lobanova Shchel (L1–L6), Vodopadnaya Shchel (V1–V10) (Gorbunov, Lukyanov, 2020; Gorbunov et al., 2020). The ecological and geographical characteristics of the places where grape plants were found are presented in Table 2.

**Table 2.**
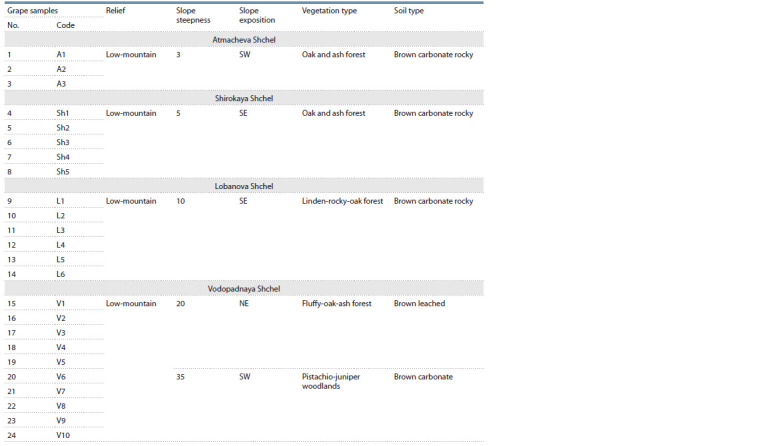
Ecological and geographical characteristics of the habitats of the analyzed wild-growing vines, Utrish Nature Reserve

DNA analysis of the found grape samples revealed different levels of polymorphism in the studied 15 loci – the number of identified alleles in the sample varied from 5 (VVMD25, VVMD27 and GF09-46) to 18 (UDV305) and amounted to an average of 8 alleles per locus (Table 3). The mean observed heterozygosity (Ho = 0.664) was lower than expected (He = 0.712). DNA marker analysis using VVIb23 showed that all wild vines were dioecious. Among the nine microsatellite loci, the data on polymorphism of which are used for DNA fingerprinting of grape genotypes, the most polymorphic was VVS2 (10 alleles were determined), the least – VVMD25, VVMD27 (5 types of alleles each) (see Table 3).

**Table 3.**
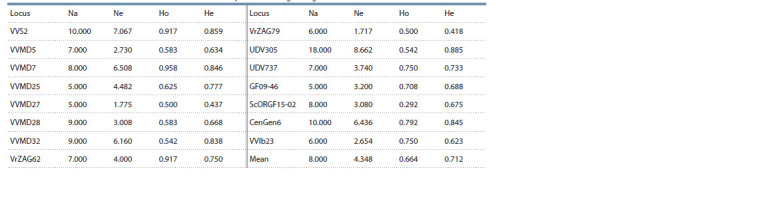
Characteristics of microsatellite loci in the studied sample of 24 wild-growing vines Notе. Na – number of different alleles, Ne – number of effective alleles, Ho – observed heterozygosity, He – expected heterozygosity

A similar situation was noted in the study of the diversity of wild vines in Armenia – the studied sample (77 samples) was also the most polymorphic in the locus of VVS2 – 13 types of alleles, and the least in VVMD25 and VVMD27 (5 and 8 alleles were identified, respectively) (Margaryan et al., 2019). At the same time, in the study of Gorislavec S.M. and co-authors (2017), in which Crimean wild-growing vines were studied, the smallest polymorphism (5 types of alleles) was revealed at the VVS2 locus (Gorislavec et al., 2017).

**Fig. 1.**
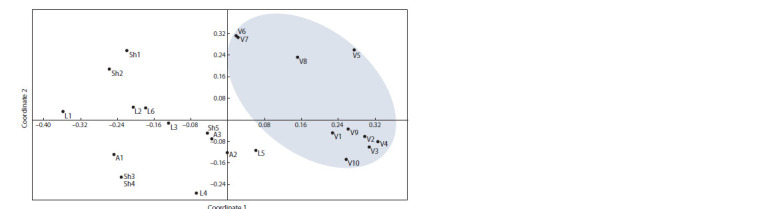
Distribution of the studied grape genotypes in the space of the main coordinates.

When distributing genotypes in the space of the main coordinates, a group of samples from territory of the Vodopadnaya Shchel can be distinguished (Fig. 1). In general, all samples from this place are localized separately from others in the space of the main coordinates, while samples V1, V2, V3, V4, V9, V10 form a separate subgroup. Samples V6 and V7 have very similar genotypes. Among other found samples, the complete coincidence of genotypes at the studied loci was determined in wild vines Sh3 and Sh4.

Analysis of samples using DNA markers linked to the resistance genes to downy mildew Rpv3, Rp10 and powdery mildew Ren3, Ren9 did not reveal resistance loci in the genotypes of wild vines. The inclusion of these DNA markers in the study was carried out in order to study the polymorphism of wild grapes at the analyzed loci, as well as a tool for the possible identification of wild-growing vines of hybrid origin. The resistance determined by Ren3, Ren9 and Rpv3 is inherited from North American grape species (V. riparia, V. rupestris, V. labruska, V. lincecumii), Rpv10 – from V. amurensis.

**Fig. 2.**
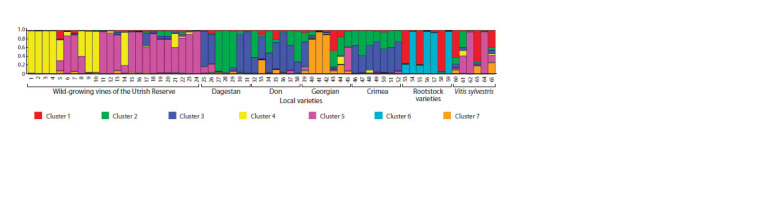
Clustering of 65 grape genotypes by origin. The vertical axis denotes the probability of assigning each genotype to putative clusters, indicated by different colors. Genotypes: 1 – V1, 2 – V2, 3 – V3, 4 – V4, 5 – V5, 6 – V6, 7 – V7, 8 – V8, 9 – V9, 10 – V10, 11 – L1, 12 – L2, 13 – L3, 14 – L4, 15 – L5, 16 – L6, 17 – Sh1, 18 – Sh2, 19 – Sh3, 20 – Sh4, 21 – Sh5, 22 – А1, 23 – А2, 24 – А3, 25 – Agadai, 26 – Rish baba, 27 – Tavlinskiy pozdniy, 28 – Hatal baar, 29 – Khop khalat, 30 – Sarakh, 31 – Khatmi, 32 – Varyushkin, 33 – Mushketnyi, 34 – Sibirkovyi, 35 – Efremovskiy, 36 – Shilokhvostyi, 37 – Tsimlyanskiy chernyi, 38 – Shampanchik bessergenevskiy, 39 – Tsitska, 40 – Aleksandrouli, 41 – Mtsvane Kakhuri, 42 – Rkatsiteli, 43 – Saperavi, 44 – Tsolikouri, 45 – Chkhaveri, 46 – Sary kokur, 47 – Kharko, 48 – Kefesiya, 49 – Sary pandas, 50 – Shabash, 51 – Dzhevat kara, 52 – Kokur belyi, 53 – Couderc 1616, 54 – Kober 5 BB, 55 – Millardet et Grasset 101-14, 56 – Teleki 8 B, 57 – Rupestris du lot, 58 – Fercal, 59 – Paulsen 1103, 60 – Gesher Hardof (Israel), 61 – Khedhayria (Tunisia), 62 – Lambrusque Abbadia H (France), 63 – Sveni (Armenia), 64 – Sylvestris Dirmstein 2 (unknown), 65 – Sylvestris Guemuelduer 104-64 (Turkey).

DNA fingerprints of the studied wild-growing vines by 9 SSR loci standard for the identification of grape genotypes were checked for a coincidence in the catalog of grape varieties’ DNA profiles of the international database VIVC (VIVC, 2022). No coincidences were found. To analyze the genetic similarity, DNA profiles of varieties belonging to geographically close regions of viticulture, where there are local varieties, some of which may have originated from wild grapevines growing earlier in these territories, were included into the research. For comparison, the identified DNA profiles of V. sylvestris genotypes from different geographical locations (Israel, Tunisia, France, Armenia, Turkey) presented in VIVC were also used. The study also included a group of rootstock varieties of complex interspecific origin with the largest share of the genetic contribution of North American grape species in order to exclude the presence of rootstock varieties among the found wild vines, which are characterized by high adaptability to various abiotic and biotic stress factors. The number of clusters equal to 7 (K=7) was used for Bayesian analysis. The results of the analysis are shown in Figure 2

The data obtained during the analysis allowed us to identify certain patterns of clustering of samples, relative to their origin. The fourth and fifth clusters mainly include samples selected from wild grapevines of the Utrish Nature Reserve. The exceptions are three genotypes of the grape subspecies Vitis

vinifera L. subsp. sylvestris (Gmelin) (V. sylvestris) – Khedhayria (Tunisia) (61), Lambrusque Abbadia H (France) (62) and Sylvestris Dirmstein 2 (64), and one local Georgian variety Chkhaveri (45) belonging to cluster 5 with a probability not exceeding 50 %. It is worth noting that the three studied wild grape vines (5, 8 and 21) were not unambiguously assigned to cluster 4 or 5. In turn, clusters 2, 3 and 7 include all the local varieties presented in the study: of Dagestan, Don, Georgian and Crimean origin. At the same time, cluster 7 is typical for three varieties of Georgian origin – Alexandrouli (40), Mtsvane Kakhuri (41) and Rkatsiteli (42); in other cases, the probability of its contribution to the genotypes is not significant. With the exception of the three varieties mentioned above, representatives of the local gene pool are distributed between clusters 2 and 3 with varying degrees of confidence. The sixth cluster was formed by a number of genotypes of rootstock varieties, the minor contribution of this cluster was also revealed only in varieties from this sample. Cluster 1 includes some rootstock varieties, part of the V. sylvestris genotypes and one Georgian variety Saperavi (43).

## Conclusion

Based on the above, we can conclude that the samples of wild grapes of the Utrish Nature Reserve selected during the expedition are represented by two hypothetical populations (expressed as clusters 4 and 5). There are transitional forms between the two populations. And if the first nominal population (cluster 4) is localized on the Vodopadnaya Shchel territory, then representatives of the second population (cluster 5) are found at all expeditionary points of sampling of plant material. A genetic relationship was also established between the second nominal population and some genotypes of V. sylvestris and the Georgian variety Chkhaveri (45), with varying degrees of probability included in cluster 5.

Thus, it can be assumed that at least part of the genotypes found in the Utrish Nature Reserve are close to the genotypes of the subspecies V. sylvestris presented in the VIVC international database. Samples from the hypothetical first population (primarily localized – Vodopadnaya Shchel) are genetically different from other vines and are predominantly allocated to cluster 4. An insignificant contribution of cluster 4 was noted in the genotypes from the gene pool of Georgian and Crimean local varieties (44 –Tsolikouri, and 48 – Kefesia), and also in two accessions of V. sylvestris (61 – Khedhayria (Tunisia), 65 – Sylvestris Guemuelduer 104-64 (Turkey)). The genetic contribution of American rootstock varieties to the wild grape population of the Utrish Nature Reserve has not been identified.

## Conflict of interest

The authors declare no conflict of interest.
